# Application of Polyacrylic Hydrogel in Durability and Reduction of Environmental Impacts of Concrete through ANN

**DOI:** 10.3390/gels8080468

**Published:** 2022-07-26

**Authors:** Kang Peng, Longliang Wu, Yousef Zandi, Alireza Sadighi Agdas, Ali Majdi, Nebojsa Denic, Aleksandar Zakić, Ahmed Abdel Khalek Ebid, Mohamed Amine Khadimallah, H. Elhosiny Ali

**Affiliations:** 1 School of Resources and Safety Engineering, Central South University, Changsha 410083, China; pengkang@cqu.edu.cn; 2 Bureau Public Works of Shenzhen Municipality, Shenzhen 518031, China; 3 Department of Civil Engineering, Tabriz Branch, Islamic Azad University, Tabriz 51579, Iran; zandi@iaut.ac.ir; 4 Ghateh Gostar Novin Company, Tabriz 51579, Iran; alireza.sadighi.agdas@gmail.com; 5 Department of Building and Construction Technologies Engineering, Al-Mustaqbal University College, Hillah 51001, Iraq; alimajdi@mustaqbal-college.edu.iq; 6 Faculty of Sciences and Mathematics, University of Priština, 38220 Kosovska Mitrovica, Serbia; nebjsadenic@pr.ac.rs; 7 Faculty of Mathematics and Computer Science, ALFA BK University, 11070 Belgrade, Serbia; aleksndarzakic@alfa.edu.rs; 8 Structural Engineering and Construction Management, Faculty of Engineering, Future University in Egypt, New Cairo 11745, Egypt; ahmed.abdelkhaleq@fue.edu.eg; 9 Civil Engineering Department, College of Engineering, Prince Sattam Bin Abdulaziz University, Al-Kharj 16273, Saudi Arabia; m.khadimallah@psau.edu.sa; 10 Laboratory of Systems and Applied Mechanics, Polytechnic School of Tunisia, University of Carthage, Tunis 1054, Tunisia; 11 Advanced Functional Materials & Optoelectronic Laboratory (AFMOL), Department of Physics, Faculty of Science, King Khalid University, P.O. Box 9004, Abha 61413, Saudi Arabia; hibrahim@kku.edu.sa; 12 Research Center for Advanced Materials Science (RCAMS), King Khalid University, P.O. Box 9004, Abha 61413, Saudi Arabia; 13 Physics Department, Faculty of Science, Zagazig University, Zagazig 44519, Egypt

**Keywords:** hydrogel, void space, concrete, nano-silica, ELM-ANFIS

## Abstract

While adding superabsorbent polymer hydrogel particles to fresh concrete admixtures, they act as internal curing agents that absorb and then release large amounts of water and reduce self-desiccation and volumetric shrinkage of cement that finally result in hardened concrete with increased durability and strength. The entrainment of microscopic air bubbles in the concrete paste can substantially improve the resistance of concrete. When the volume and distribution of entrained air are adequately managed, the microstructure is protected from the pressure produced by freezing water. This study addresses the design and application of hydrogel nanoparticles as internal curing agents in concrete, as well as new findings on crucial hydrogel–ion interactions. When mixed into concrete, hydrogel particles produce their stored water to power the curing reaction, resulting in less volumetric shrinkage and cracking and thereby prolonging the service life of concrete. The mechanical and swelling performance qualities of the hydrogel are very sensitive to multivalent cations found naturally in concrete mixes, such as aluminum and calcium. The interactions between hydrogel nanoparticles and alkaline cementitious mixes are described in this study, while emphasizing how the chemical structure and shape of the hydrogel particles regulate swelling behavior and internal curing efficiency to eliminate voids in the admixture. Moreover, in this study, an artificial neural network (ANN) was utilized to precisely and quickly analyze the test results of the compressive strength and durability of concrete. The addition of multivalent cations reduced swelling capacity and changed swelling kinetics, resulting in fast deswelling behavior and the creation of a mechanically stiff shell in certain hydrogel compositions. Notably, when hydrogel particles were added to a mixture, they reduced shrinkage while encouraged the creation of particular inorganic phases within the void area formerly held by the swelled particle.

## 1. Introduction

**Figure 1:**
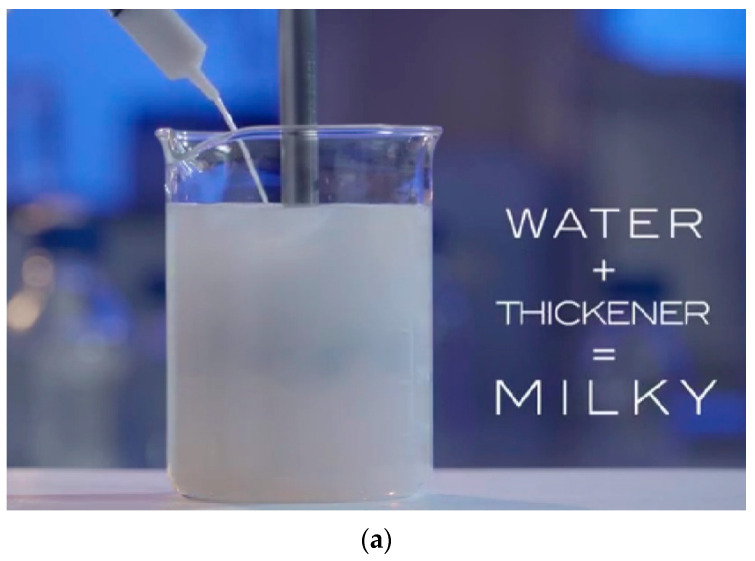

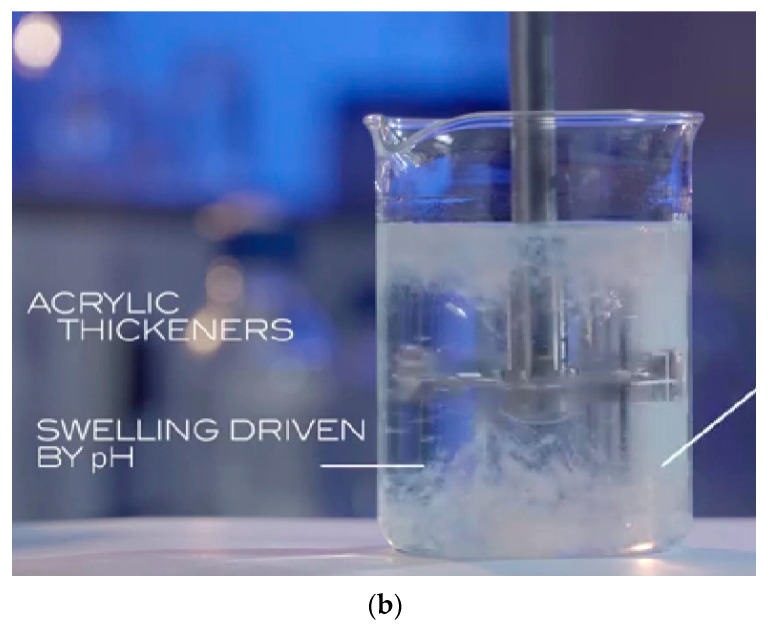
A range of chemical additives have been added to improve the general operation of ordinary polyacrylic hydrogel. (**a**) Combining of water, acrylic thickener, and adding PH adjustment, then mix for few minutes, (**b**) Swelling that was derived by PH.

Portland cement is the most frequently used construction material in the world, and its production accounts for 5–10 percent of the globe’s total yearly carbon dioxide emissions [1,2,3,4]. In the US, 92 million metric tons of concrete were manufactured in 2015, providing $10.6 billion to state revenues [5]. Therefore, concrete is a vast international sector, and there is significant opportunity for the adoption of a broad variety of material science technologies that enhance the effectiveness and sustainability of concrete materials [6,7,8,9]. Such material developments would in turn lower the need for maintenance and repair of concrete pavement and infrastructure over time, resulting in significant environmental and economic savings [10,11,12,13,14]. High performance concrete (HPC) is an innovative alternative to traditional concrete that is more durable and stronger owing to its reduced porosity and fewer CO_2_ emissions [1,15,16,17,18,19,20]. HPC may easily attain compressive strengths as high as 110 MPa, as opposed to the more typical 30–40 MPa [21]. Reduced porosity and a disconnected pore network render HPC highly resistant to corrosive fluid penetration, making it very durable, even in severe conditions [22,23,24,25]. Due to its decreased permeability, HPC has generally strong fire resistance; nonetheless, the narrower pore network might produce explosive spalling at high enough temperatures [26,27,28,29,30,31]. Such spalling may be minimized by including soft polymers, such as crushed rubber, into the concrete, since they impart a greater elastic modulus [32]. Self-desiccation is the outcome of high Portland cement content compared to free water (low water-to-cement ratio) in high-performance concrete. Water is used with Portland cement to produce calcium silicate hydrate (CSH), the inorganic binder from which concrete derives its compressive strength [33]. Significant inward Laplace pressures are increased in HPC mixtures when water is drained and consumed from the hydrated cement network’s tiniest pores [34,35,36,37,38]. These stresses are sufficient to generate autogenous shrinkage, which may lead to early-age cracking and structural failure, particularly if the concrete is restricted by reinforcements, forms, or other concrete [39]. During the curing phase, more water may be added from external sources to limit self-desiccation and counterbalance a portion of this early-age shrinkage. Due to the tight microstructure and poor permeability of HPC, it is difficult for water from the outside to permeate deeply into the matrix of hardening cement. Internal cure approaches provide a remedy to this HPC self-desiccation issue. Recent research has focused on the utilization of covalently cross-linked superabsorbent polymer (SAP) hydrogels [40] for internal curing. These hydrogels may capture and hold fluids up to a few hundred times their dry weight [41]. Hydrogels were observed to prevent autogenous shrinkage in HPC, and while some studies have reported a reduction in early age strength following the addition of hydrogels, the strength did rebound to control levels with adequate time [42]. The capacity to alter the swelling response, the mechanical response, and the size of hydrogels may potentially result in hydrogels conferring several advantages to concrete. Hydrogels may be added dry to the cement admixture, and very low quantities have been shown to be helpful in minimizing HPC shrinkage (often less than 2 percent by weight of cement), making them more desirable than pre-soaked lightweight aggregates [43,44]. A deficiency in the study on polymer hydrogels for internal curing is the widespread use of proprietary hydrogels with undisclosed chemical compositions; typically, not even the monomers utilized to synthesis the hydrogels are revealed. The underlying assumption is that hydrogels inside solid materials are chemically inert. Recent research [43,44,45,46,47,48,49] has shown that when hydrogels are to be employed as internal curing agents for concrete, a great deal of consideration should be given to their chemical composition as well as the technique for measuring their swelling capacity. Gels 2017, 3, 45 2 of 18 HPC includes an overabundance of Portland cement compared to free water (low water-to-cement ratio), resulting in the self-desiccation phenomena [50,51,52,53,54]. Water is used by Portland cement to produce calcium silicate hydrate (CSH), the inorganic binder from which concrete derives its compressive strength [55]. During the curing phase, more water may be added from external sources to limit self-desiccation and counterbalance a portion of this early-age shrinkage. Due to the tight microstructure and poor permeability of HPC, it is difficult for water from the outside to permeate deeply into the matrix of hardening cement. Internal curing approaches provide a remedy to this HPC self-desiccation issue. Recent research has focused on the utilization of covalently cross-linked superabsorbent polymer (SAP) hydrogels [56] for internal curing. These hydrogels have the capacity to absorb and hold fluids up to several hundred times their dry weight [41]. Hydrogels have been observed to prevent autogenous shrinkage in HPC, and while some studies have suggested a reduction in early age strength following the addition of hydrogels, the strength did rebound to control levels with adequate time [39,57,58]. The capacity to alter the mechanical response, swelling response, form [59], and size of hydrogels may potentially result in hydrogels conferring several advantages to concrete. Hydrogels may be added dry to the cement mixture, and very low quantities have been shown to be helpful in minimizing HPC shrinkage (often less than 2 percent by weight of cement), making them more desirable than pre-soaked lightweight aggregates [60]. A deficiency in the study on polymer hydrogels for internal curing is the widespread use of proprietary hydrogels with undisclosed chemical compositions; typically, not even the monomers used to synthesis the hydrogels are revealed. The underlying assumption is that hydrogels inside solid materials are chemically inert. Figure 1 shows a range of chemical additives added to improve the general operation of ordinary polyacrylic hydrogel.

**Figure 2:**
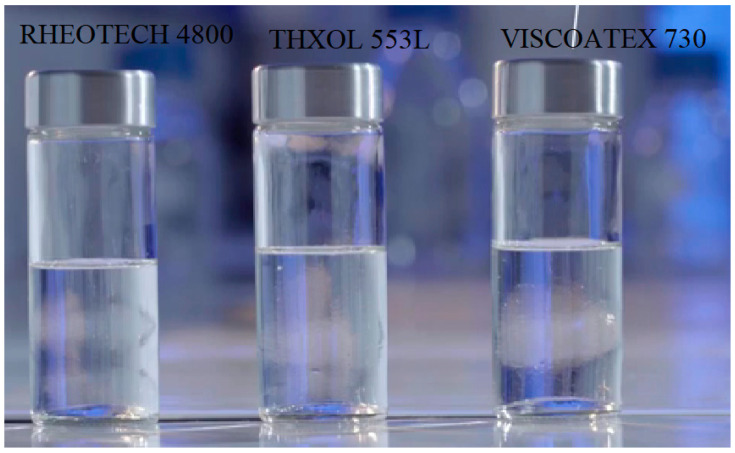
Three types of gel structures.

The tight microstructure produces a sturdy and long-lasting structure [26] with minimal environmental impact [32]. The biggest technical problem with HPC is autogenous/volumetric shrinkage, which occurs initially in the curing process and subsequently leads to the production of greater porosity, micro cracks, and a decrease in the general strength [55]. The amount of shrinkage increases due to insufficient cement hydration because of the lack of water to drive curing, causing the mix to self-desiccate [33]. Negative capillary pressure inside the aqueous fluid in the combination (dubbed ‘pore fluid’) causes compressive stresses within the system, eventually resulting in the destruction of the cement microstructure [33]. Internal curing agents that efficiently replace the water supply within the mix and assist in cement hydration can be used to overcome the deficiency of water within the system [61,62,63,64,65,66,67]. For the internal curing of HPC, superabsorbent polymers (SAPs) have become popular. SAP hydrogel particles take up water and expand to a thousand times their initial size [40]. As a result, SAPs can supply a regulated discharge of a large water reservoir for the use of HPC internal curing [56]. Thus, autogenous shrinkage and micro cracking in concrete are successfully decreased, while a very tough and durable concrete is formed [33]. There are other advantages to employing SAP particles as internal curing agents for concrete, out of which some are presently being investigated: concrete freeze or thaw resistance can be improved by controlling the size and shape of the pore systems [68], thermal expansion can be reduced [69], and fractures can be loaded to facilitate healing of fractures [70]. The utilization of hydrogel particles also allows for greater supervision over the rheological characteristics of fresh concrete [71] as well as a decrease in fire-caused spalling. Figure 2 shows three types of gel structures, namely, RHEOTECH 4800, THXOL 553L, and VISCOATEX 730.

**Figure 3:**
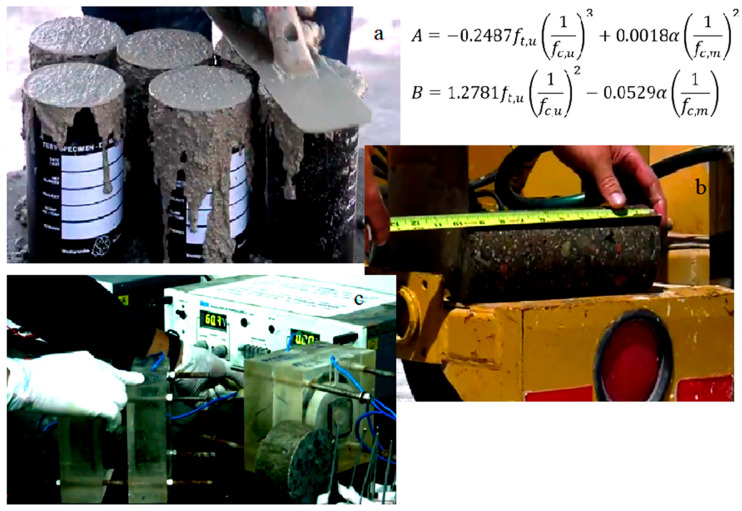
Durability test: deicer scaling of the concrete surface (**a**) Durability test, deicer scaling of the concrete surface, (**b**) Measure of the size and weight, (**c**) Cut to the pieces of 50 mm × 100 mm.

**Figure 4:**
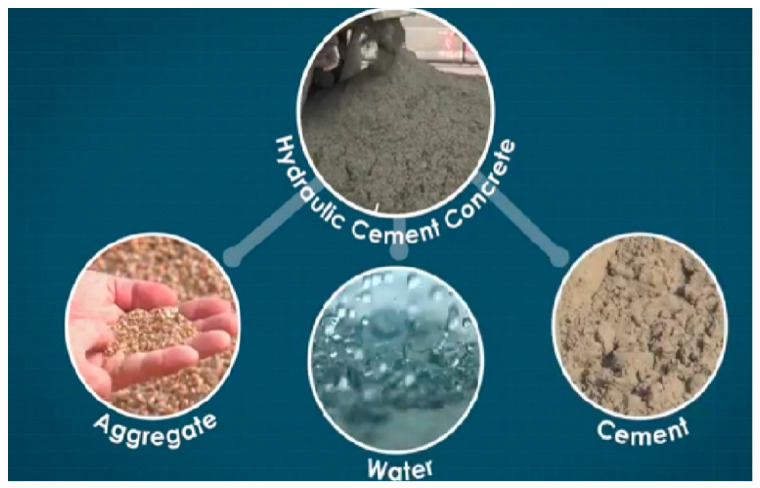
Three main components of cement-based concrete.

Portland cement is the most frequently used construction material on the planet, with its manufacture accounting for five to ten percent of global yearly CO_2_ production [1]. In 2015, ninety-two million metric ton of concrete were manufactured in the United States, generating 10.6 billion dollars in state revenue [5]. As a result, concrete is a vast global sector with enormous promise for incorporating a wide range of material science approaches to improve sustainability as well as performance of concrete substances [4,35,36,37,38,72]. These material advancements would then gradually lower the demand for concrete infrastructure as well as pavement replacement and repair, resulting in substantial environmental and economic advantages. Owing to its smaller porosity and fewer CO_2_ emissions, HPC is a more durable and strong replacement for traditional concrete [73]. In comparison to the more normal compressive values of 30 to 40 MPa, HPC may easily produce compressive strengths as great as 110 MPa [21]. HPC is resilient to corrosive fluid intrusion due to its low porosity and unconnected pore network, making it exceptionally stable even in severe conditions [22]. HPC has excellent fire resistance because of its decreased permeability, yet the narrower pore network may produce catastrophic spalling when adequately extreme temperatures are attained [26]. The inclusion of soft polymers, such as crushed rubber can help to prevent spalling by giving the concrete a greater elastic modulus [32]. HPC comprises an overabundance of Portland cement in comparison to free water (it has a small water to cement ratio), resulting in self-desiccation [74,75,76,77]. Water is used by Portland cement to produce calcium silicate hydrate (also called CSH), which is an inorganic binder that gives concrete compressive strength [55]. As water is used and removed from the small holes in the hydrated cement network in HPC mixes, large inward Laplace pressures occur [34]. These pressures are significant enough to generate autogenous shrinkage, a bulk volumetric collapsing of the system that can result in early age cracks and failure of the structure, particularly if the concrete is restricted against reinforcements, forms, or even other concrete [17,39,78,79,80,81,82,83]. Extra water can be given externally during the curing stage to prevent self-desiccation and counterbalance part of this early age shrinkage through the use of wet blankets, hoses, or sprinklers. External water cannot delve deep into the curing cement matrix because of the thick microstructure and poor permeability of high performance cement. Internal cure approaches provide a resolution to the self-desiccation challenge in high performance concrete. These hydrogels may take in and hold fluid even a hundred times their dry weight [41]. Although some researchers have observed a decline in the early-age strength upon introducing hydrogels, strength recovered to control levels with enough time [42]. Controlling the mechanical reaction, swelling response, form [84], and size of the hydrogels could lead to many benefits for concrete. Hydrogels can be introduced dry to the cement mix and have been shown to reduce high performance concrete shrinkage (typically around two percent by weight of cement), making them more appealing to utilize than presoaked lightweight aggregates [85]. During the plastic and rigid states of concrete and absorption and desorption water [86,87,88,89,90,91]. the dispersed components contribute internal water to the cement gel, accelerating the hydration stage in cement-based substances. Practitioners and academics have widely used two main substances, lightweight aggregate (LWA) and superabsorbent polymer (SAP), to meet internal curing in concrete sections. Superabsorbent polymers are polymers that are cross linked and can take in and discharge significant amounts of water and can be used in a variety of concrete technological uses. SAPs’ swelling rate and ability to change relying on polymer type, chemical makeup, and size are key properties [39]. SAP is described as a smart material that is able to alter its characteristics in a regulated manner in response to an outside effect. Figure 3 shows hydrogel rheology. Elastic solids σ=Gγ stress and strain are in phase, and viscous fluid σ=ηγ stress and strain are out of the phase. Figure 3 shows a durability test and deicer scaling of the concrete surface (a); these distresses can be mitigated by proper mixture design. The three main components of cement-based concrete are shown in Figure 4.

According to the literature, the swelling ratio of SAP in deionized water can be larger than 500 g/g, whereas in a traditional concrete pore solution, it can be as low as 10 to 20 g/g. Whenever the external humidity levels drop, the enlarged SAP acts as a flow obstacle, slowly releasing absorbed water [15,92,93,94,95,96,97]. Apart from tangible uses, SAP has been seen in meat packing, pharmaceutical uses such as delivery of drugs, personal hygiene products such as diapers, biomedical products such as band aids, trash solidification, agricultural uses for the conditioning of soil, and water barrier tapes for underwater water pipes [98]. SAPs are primarily used as internal curing agents in concrete technology to prevent autogenous shrinkage in small water to cement ratio mixes, self-healing, rheological management, and frost prevention. RILEM STAR-225 produced a futuristic paper on SAP use in concrete (2012). The advantages of SAP over alternative internal curing agents, such as LWA, lay in the small amount of SAP required to provide internal curing water and enhance concrete’s mechanical and freshness qualities. It could be used as an air entraining substance in concrete to enhance air content and hence raise freeze thaw resistance [99,100,101,102]. The water absorption rate can also be calculated analytically. According to Jensen and Hensen (2001), the benefits of SAP as an internal curing agent include preventing self-desiccation, reducing cracks, increasing hydration and longevity, and lowering permeability [39]. They also stated that adding SAP to the cement matrix improves the composite concrete’s tensile strength and toughness.

**Figure 5:**
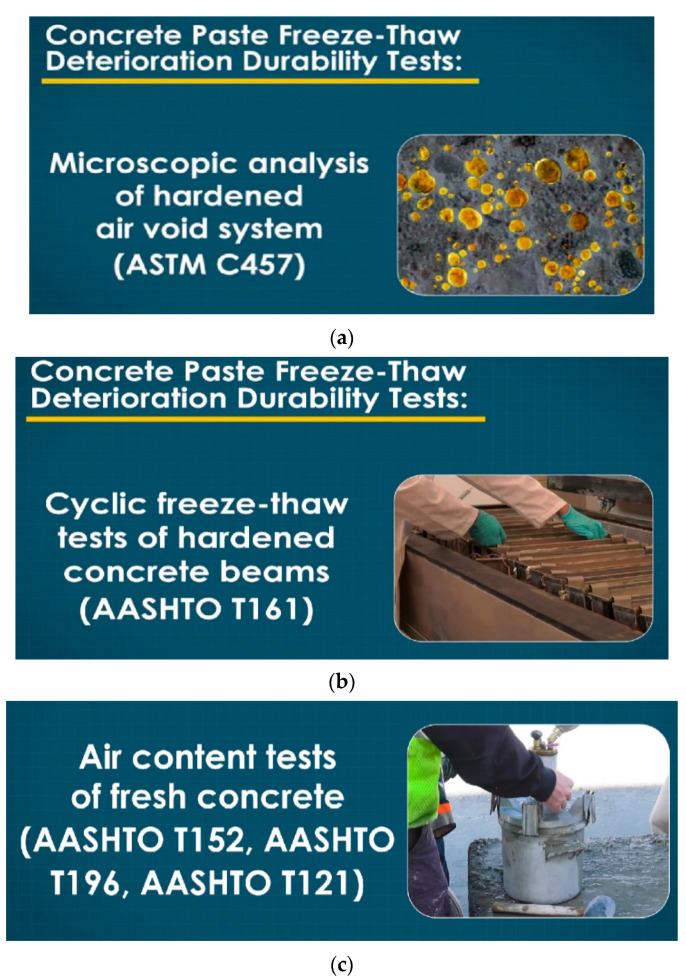
Durability test processing, (**a**) Microscopic analysis of hardened air void system, (**b**) Cyclic freeze thaw tests of hardened concrete, (**c**) Air content of fresh concrete.

The technique of limiting the volume of voids in aggregate mixtures for attaining the appropriate qualities of fresh and hardened product can be defined as mix proportioning of materials made up of cement, e.g., paste such as cement and water, as well as concrete [103,104,105,106,107]. The use of particle packing systems to estimate the void ratio as well as packing density of concretes might supply instruments for improving the performance of hardened and fresh substances by decreasing the quantity of cement and free water and increasing the number of solids. Nevertheless, the designer’s challenge is to balance the mix elements so that the solid mix components have a minimal void ratio and maximal packing density while maintaining acceptable operability [92,108,109,110,111,112]. Figure 5 shows the three phases of durability test processing: microscopic analysis of the hardened air void system, cyclic freeze thaw tests of hardened concrete, and air content of fresh concrete.

### Objective of Study

**Figure 6:**
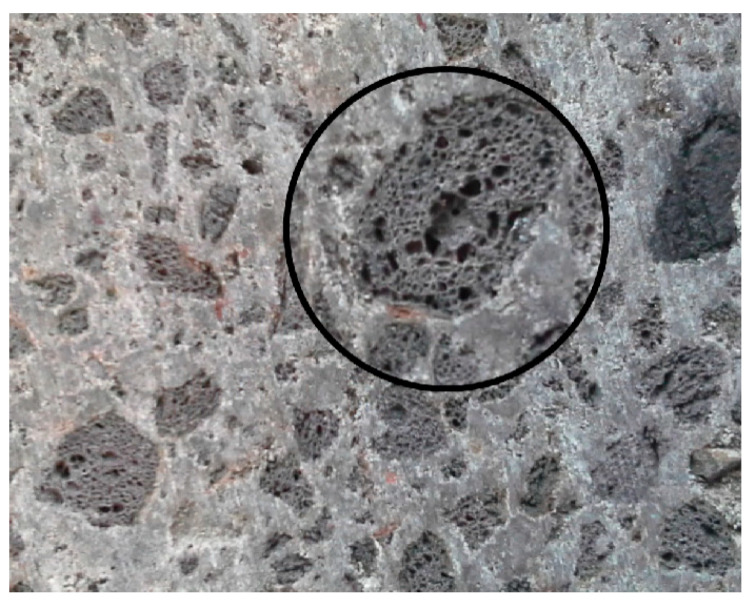
Microscopic image of AASHTO T152 fresh concrete.

In this work, packing principles were used to estimate the paste volume required to fill aggregate void space in order to estimate the void ratio of various aggregates. Lastly, theoretical approaches for cement content, slump, and compressive strength were established. Subsequently, the void content density and fineness modules of the aggregate were estimated, the slump cone test was employed to estimate the needed volume of cement paste, and the concrete compressive strength was calculated. ANN was utilized to improve the accuracy of the test by increasing the analysis duration and decreasing the ratio of error. Figure 6 shows the hardened air void system (ASTM C457). Figure 6 shows a microscopic image of AASHTO T152 fresh concrete.

## 2. Methodology

### 2.1. Hydrogel Chemistry

Polyelectrolyte molecules are covalently cross linked to produce a three-dimensional polymer network, which is employed as an internal curing agent. Due to the presence of very alkaline pore fluids (pH greater than 12), hydrogel particles are not chemically inactive in cement mixes [113]. As pKa of acrylic acid is roughly 4.5, the carboxylic acid (COOH) functional groups in hydrogel particles that contain acrylic acid deprotonate in alkaline conditions, forming anionic COO– moieties in the polymer network and enabling substantial quantities of water to be soaked up via ion dipole interplay. Since the anionic network results in a higher concentration of free counter ions inside this hydrogel particle in comparison to the surrounding fluid for maintaining electro neutrality inside the system, the primary method of hydrogel particle swelling could be related to the creation of a chemical potential gradient between the surrounding fluid and particle. This gradient causes an osmotic pressure gradient, which causes water and other ions in solution to diffuse into the hydrogel particle. The particle will expand till the overall osmotic pressure is 0. Cations in the aqueous fluid such as sodium ions, aluminum ions, and calcium ions will be electrostatically drawn to the COO– moieties and produce ionic complexes that essentially function as crosslinks inside the polymer network, lowering the hydrogel’s equilibrium absorbency and leading to polymer network breakdown. As specific hydrogel compositions (inclusive of some commercially available substances) have been discovered to show great sensitivity to the mono and multivalent cations that naturally occur in pore fluids based on mix age, there is a wealth of research in the concrete materials field to precisely measure the absorption and desorption behavior of hydrogel particles in cement pore fluids. Ion-induced deswelling is improved as the anionic character of the hydrogel is enhanced, e.g., by increasing the concentration of acrylic acid in the network due to the greater availability of anionic sites in the polymer network that are able to complex with counter ions in solution.

### 2.2. Hydrogel–Cement Interactions

Hydrogel particles are often utilized in extremely small quantities when added with cement (generally only 0.2 percent by weight of dry cement). In the lab, cement and hydrogel are combined dry, the needed volume of superplasticizer and water are introduced, and the mix is vacuum or hand combined (or even machine-mixed at the field). A vacuum mixer can provide more consistent mixing while also assuring that any porosity is caused by hydrogel particles and capillary water rather than non-uniformity as a result of the manual mixing process. As the cement paste for HPC has a very small w/c ratio, the superplasticizer, which is usually a water reducing additive, is included to provide adequate workability. When water is added to the mix, hydrogel particles instantly swell, and by the time the cement mix is applied, hydrogel particles are probably fully expanded (such as in a cast). The interplay between hydrogel particles and cement paste is of primary importance because hydrogel particles are needed for maintaining adequate hydration of the cementitious matrix; coarse aggregate (i.e., rock pieces) and fine aggregate (i.e., sand) have been excluded from the illustrative example for convenience. It is also assumed that a comparable hydration process would take place in concrete as well as mortar specimens. Concrete mixture data were taken from [114] (Table 1).

### 2.3. Material

**Figure 7:**
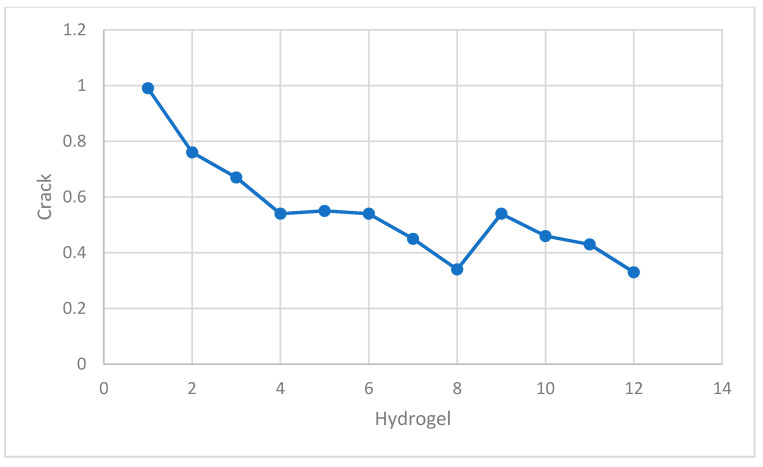
The application of hydrogel in concrete reduces shrinkage and cracking.

**Figure 8:**
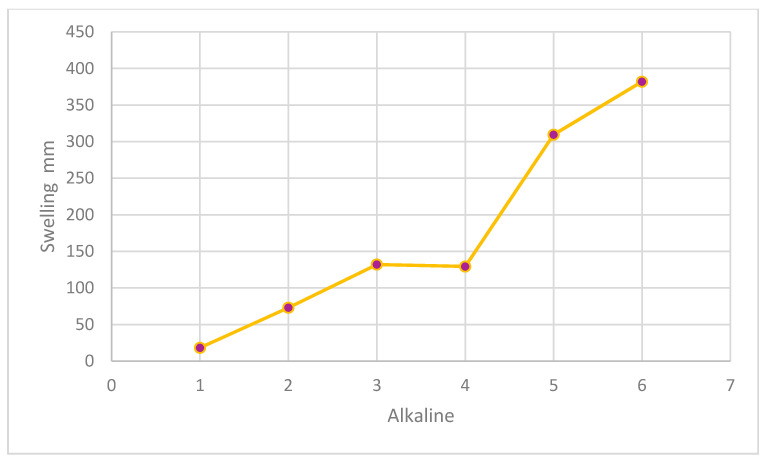
Interactions of alkaline cementitious mixes and hydrogel nanoparticles on the swelling and curing behavior increase the elimination of voids in the admixture.

**Figure 9:**
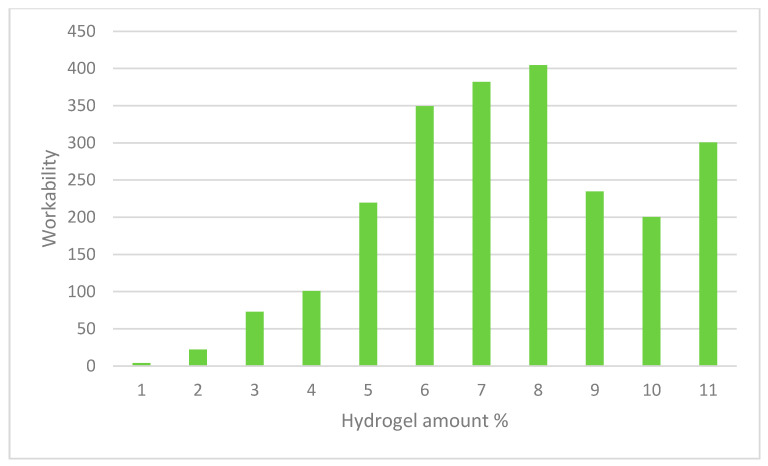
The application of hydrogel stabilizes the workability level irrespective of the w/cm by means of a minimal paste to void ratio.

ASTM C29 was used to determine the total aggregate void content. The amount of oven dry coarse and fine aggregate needed to maintain the chosen aggregate ratio was calculated. The fine and coarse aggregates were combined together in a pan using a scoop. The blended mixed aggregate was layered thrice in a 0.33 cubic foot vessel, with each layer being rodded twenty-five times. The void content was determined using the bulk density and aggregate relative density measurements. The test was carried out thrice with different batches each time. The mixed aggregate’s average void content was determined to be 25.4 percent. Many various molarity aluminum solutions were created, and gravimetric measurements were made to further explore the impacts of aluminum on swelling responses along with hydrogel mechanics. Lastly, the elastic modulus of hydrogels expanded in 0.025 M aluminum solution was calculated as a function of time. The kinetics of swelling was also altered. At a concentration of 0.005 M, all hydrogels exhibited a brief peak of swelling (5 min) followed by consistent deswelling for the rest of the experiment. Table 2 shows mixture proportions for pastes with and without hydrogels across all samples. WRA is percent by weight of cement. Q is swelling ratio obtained in pore solution at 4 h after immersion following the study [115]. Figure 7 shows that the application of hydrogel in concrete reduces shrinkage and cracking. Figure 8 also shows that the interactions of alkaline cementitious mixes and hydrogel nanoparticles on the swelling and curing behavior increase the elimination of voids in the admixture. Figure 9 shows that the application of hydrogel stabilizes the workability level, irrespective of the w/cm by means of a minimal paste to void ratio.

### 2.4. Artificial Neural Network (ANN)

**Figure 10:**
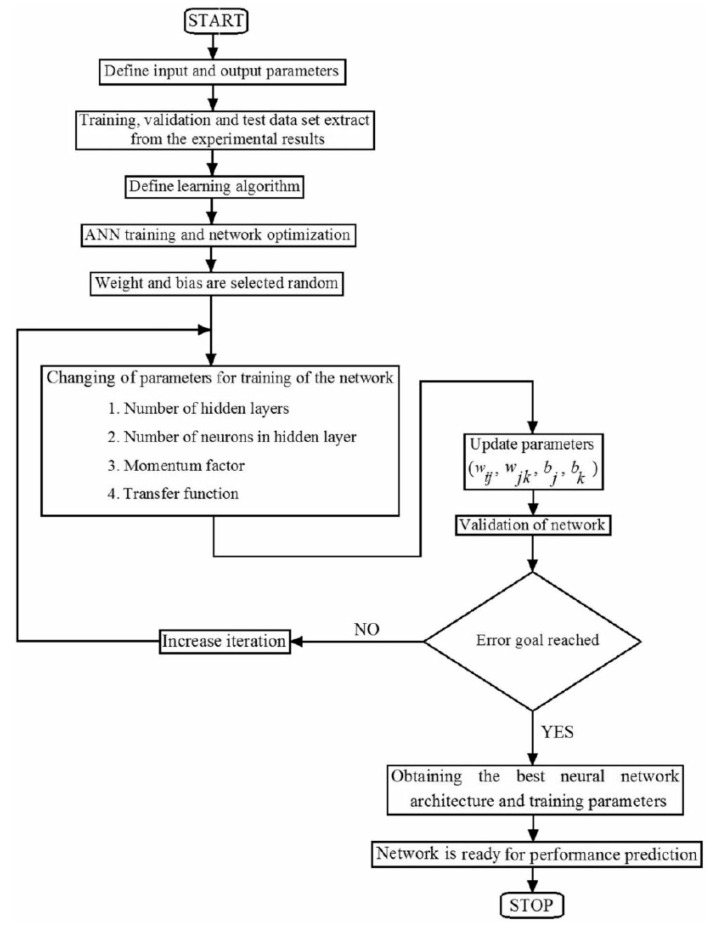
ANN flowchart.

Artificial neural networks (ANNs) have lately received a lot of attention as a novel way of processing data [116,117,118,119,120]. ANN attempts to mathematically simulate biological brain neural networks [121,122,123,124,125]. The brain is a vast-scale system that connects a huge number of neural cells known as neurons. The brain has numerous interesting properties, such as parallel information processing, learning capacity, and self-organization capabilities, to name a few [46,47,108,126,127,128]. The ANN is a brain simulation that links numerous nonlinear or linear neuron models and analyzes data in a distributed, parallel fashion. ANN can do operations at a considerably faster rate due to its highly parallel feature [61,62,63,64,129]. Furthermore, ANN has a lot of intriguing and appealing aspects (Figure 10). As a result, ANN can adjust to changes in data by learning input signal properties [130,131,132,133]. An ANN may learn mapping among input and output space and create an associative memory that fetches the proper output when given the input and generalizes when given additional inputs [50,51,52,53,72,134]. ANN can also conduct functional estimation and signal-filtering activities that are beyond the capabilities of optimal linear approaches due to their nonlinear character [87,88,89,90]. McCulloch and Pitts devised a neuron model and demonstrated its usefulness in a logical operation system. The Rosenblatts perceptron sparked a lot of interest for its conceptual ease. The perceptron should not be employed for the use of sophisticated logic functions, according to Pviinsky and Papert [135]. A complete study of notable ANN has been given by Richard P. Lippmm [136]. Many researchers have claimed that the feed forward model of multilayered perceptron (MLP) produces positive outcomes in many settings. MLP employs the back propagation algorithm (also known as BPA). The capacity of an ANN [137,138,139,140,141] to acquire the broader relationship among variables is the focus of the ANN testing presented in this study. A vast number of basic processing elements (PE) known as neurons make up the ANN. Every PE has a large number of outputs and inputs. Connection weights are the output paths of a PE. As every link has a corresponding weight, those weights modify the signals on the input lines to the processing device. Summation is used to integrate these weighted signals. An activation function modifies the combined signal before passing it to the processing element’s output path [142].

## 3. Result and Discussion

### 3.1. Model Performance Indicators

For this work, three regression formulas for the root mean square error (*RMSE*), the coefficient of determination (*R*^2^), and the mean absolute error (*MAE*) were applied for verification of the exactness of values in the testing and training phases of the model for foreseeing compressive strength and durability of concrete and assessing voids of concrete while using the hydrogel. The following is how the indices are calculated:

(1) $$r=\frac{N(\sum_{i=1}^{N}O_{i}\cdot P_{i})-(\sum_{i=1}^{N}O_{i})\cdot (\sum_{i=1}^{N}P_{i})}{\sqrt{\left(N\sum_{i=1}^{N}O_{i}^{2}-{\left(\sum_{i=1}^{N}O_{i}\right)}^{2})\cdot (N\sum_{i=1}^{N}P_{i}^{2}-{\left(\sum_{i=1}^{N}P_{i}\right)}^{2}\right)}}$$

(2) $$R^{2}=\frac{{\left[\sum_{i=1}^{N}\left(O_{i}-\bar{O}\right)\cdot \left(P_{i}-\bar{P}\right)\right]}^{2}}{\sum_{i=1}^{N}\left(O_{i}-\bar{O}\right)\cdot \sum_{i=1}^{N}\left(P_{i}-\bar{P}\right)}$$

(3) $$RMSE=\sqrt{\sum_{i=1}^{N}\frac{1}{N}{\left(O_{i}-P_{i}\right)}^{2\;}}$$

N = the number of training or testing samples

$O_{i}$ = observed values in sample i

$P_{i}$ = predicted values in sample i

$\underline{O}$ = the mean observed values

$\underline{P}$ = the mean predicted values

Note: *R*^2^ of 1, *RMSE* of 0, *VAF* of 100%, and *MAE* of 0 are the ideal forms in a predictive model.

**Figure 11:**
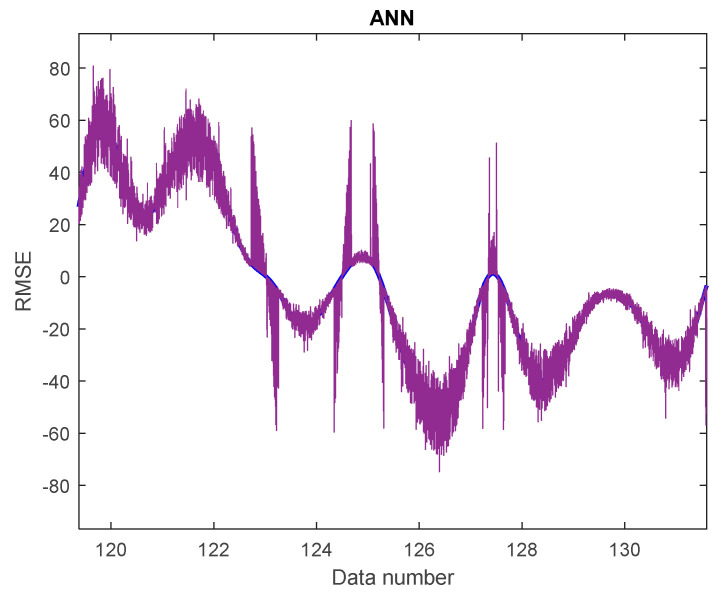
*RMSE* of ANN in test phase.

**Figure 12:**
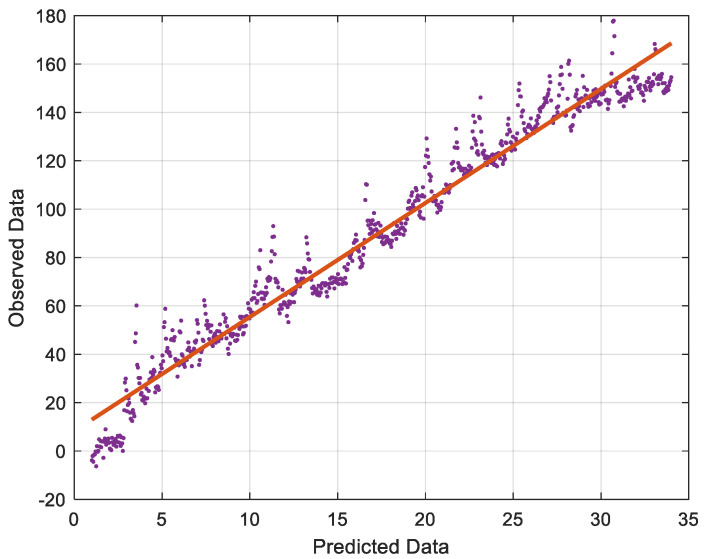
Data distribution on ANN.

Upon assessing the ANN’s included parameters, its performance in terms of previously defined performance measures was evaluated during the training and testing stages (Table 3). The goodness of fit models in the testing of half of the data were chosen as the major criteria to evaluate the performance of both models in terms of prediction accuracy. ANN has an associated *RMSE* score of 0.543. The associated *R^2^* values for ANN are 0.984. The optimum model for forecasting compressive strength and concrete durability as well as measuring voids while utilizing hydrogel was identified by contrasting values of RSQR and *RMSE*. Based on the figure, it appears that the density of color dots across the regression line is acceptable in this situation. It demonstrates ANN’s goodness. In addition, the image depicts the ANN’s *RMSE*, which demonstrates its correct performance. Going through Figure 11, there is a good correlation between the colored and blue line, showing the properness of the model in this analysis. In this figure, the horizontal axis shows the data number, and the vertical axis shows the *RMSE* values (−80 to 80). In Figure 12, the distribution of data on the regression line is represented. There is some noise on the regression line; the closer the noise is to the cross line, the more accurate the model is.

### 3.2. Experimental Analysis

Table 4 shows that the measured air content ranged from 2.5 to 4.9 percent, which was higher than the assumed value of 1.5 percent. This was an unforeseen result of not using deforming chemicals to decrease air content. The yield-adapted paste volumes for the goal 23 percent paste were on average 25.1 percent, and for the target 25 percent paste were on average 26.6 percent, due to the greater air contents. In comparison to the targets of 1.04 and 1.14, the corresponding average paste to void ratios were 1.14 and 1.20, correspondingly. To obtain equivalent slumps, the combinations with a smaller paste volume required a greater dose of HRWRA than the mixes with a greater paste volume. Nevertheless, the same dosages were needed for mixes with comparable mixing water concentrations but differing paste volumes. When examining mixes 0.55PC23 and 0.40PC25, as well as mixtures 0.55FA23 and 0.40FA25, this was noted. It shows how the concrete compositions were consistent throughout the slump measurement. Before incorporation of hydrogel, the 0.55PC23 mix appeared rough, but its inclusion increased the workability to an adequate degree. In conclusion, when hydrogel was utilized, the minimal paste to void ratio required to achieve a specified workability level remained reasonably stable irrespective of the w/cm.

## 4. Conclusions

This research investigates the relationship amongst concrete strength growth and void distribution pattern utilizing hydrogel. The void structure of the concrete section is seen as a sign of bleeding. The void volume fracture of concrete samples is estimated using an image analysis technique. Upon first look, the obtained data appeared to show a direct relationship between concrete specimen strength growth and void distribution patterns when hydrogel was not present. The inclusion of slag in the combinations, on the other hand, changed the situation inversely. We created two different hydrogel formulations and tested swelling kinetics in calcium, aluminum, and sodium solutions in this work. Since sodium ions generated electrostatic shielding inside the polymer network, the swelling capacity was lowered. Calcium and aluminum ions were capable of forming ionic complexes with the polymer network, resulting in decreased swelling capacity, deswelling of the hydrogel over time, and the creation of a mechanically rigid outer shell in the instance of trivalent aluminum ions. Concrete’s compressive strength, durability, and density were all examined. The test data were analyzed using ANN, which performed admirably. *R*^2^ and *RMSE* findings showed that ANN is a reliable technique for these analyses. Hydrogel compressive strength was studied as a function of immersion period and hydrogel chemistry, along with aluminum solution concentration. Aluminum ions seem to tightly connect with the polymer network, increase the elastic modulus (showing an increased level of crosslinking), and cannot be rinsed off with water, but calcium ions do not create persistent bridges among charged moieties on the polymer backbone. The coiling and shielding properties of aluminum form a structurally strong outer shell and hollow down the core of most acrylic acid hydrogels, indicating that aluminum ions may be harming the polymer network, but additional research is required.

## Figures and Tables

**Table 1:** Mixture constituents of the concrete specimen [114].

	M1	M2	M3	M4
Water	144	144	192	192
Cement II 42.5N	480	480	480	480
Fine aggregate (river-dredged sharp sand)	500	500	500	500
Coarse aggregate (19mm max. size)	1000	1000	1000	100
SAP (<600 µm FLOSET CC 27)	-	-	0.96	0.96
Super plasticizers CONPLAST SP 432MS	7.2	7.2	7.2	7.2
W/C ratio	30%	30%	35%	35%
Curing medium	Fresh	Marine	Fresh	Marine
	water	water	water	water

**Table 2:** Mixture proportions for pastes with and without hydrogels.

Type	Cement (kg)	Water (kg)	w/c	Hydrogels (kg)	*Q*	WRA
Control	200	70	0.35	0.4	-	0.7
17 wt% AA	200	70	0.35	0.4	22	0.7
33 wt% AA	200	70	0.35	0.4	18.2	0.7
67 wt% AA	200	70	0.35	0.4	11.7	0.7
83 wt% AA	200	70	0.35	0.4	4.3	0.7

**Table 3:** The regression test results (test phase).

Model	*RMSE*	*r*	*R* ^2^
**ANN**	0.543	0.765	0.984

**Table 4:** Mean air content of significant variables.

Independent Variables	Mean Air Content (%)	% Increase in Air Content	*p*-Value
A	5.3	8.2	<0.0001
B	6.1		
G	5.7	7.2	0.0003
L	6.1		
No	5.8	5.2	0.0033
Yes	6.1		
70	5.7	7.0	0.0003
90	6.1		

## Data Availability

Not applicable.
